# Role of the ESCRT Complexes in Telomere Biology

**DOI:** 10.1128/mBio.01793-16

**Published:** 2016-11-08

**Authors:** Anna K. Dieckmann, Vera Babin, Yaniv Harari, Roland Eils, Rainer König, Brian Luke, Martin Kupiec

**Affiliations:** a Division of Theoretical Bioinformatics (B080), German Cancer Research Center (DKFZ), Heidelberg, Germany; b Department for Bioinformatics and Functional Genomics, Institute for Pharmacy and Molecular Biotechnology (IPMB) and BioQuant, Heidelberg University, Heidelberg, Germany; c Department of Molecular Microbiology and Biotechnology, Tel-Aviv University, Tel Aviv, Israel; d Integrated Research and Treatment Center, Center for Sepsis Control and Care (CSCC), Jena University Hospital, Jena, Germany; e Network Modeling, Leibniz Institute for Natural Product Research and Infection Biology, Hans Knöll Institute, Jena, Jena, Germany; f Zentrum für Molekulare Biologie der Universität Heidelberg (ZMBH), DKFZ-ZMBH Alliance, Heidelberg, Germany; g Institute of Molecular Biology gGmbH, gefördert durch die Böhringer Ingelheim Stiftung, Mainz, Germany

## Abstract

Eukaryotic chromosomal ends are protected by telomeres from fusion, degradation, and unwanted double-strand break repair events. Therefore, telomeres preserve genome stability and integrity. Telomere length can be maintained by telomerase, which is expressed in most human primary tumors but is not expressed in the majority of somatic cells. Thus, telomerase may be a highly relevant anticancer drug target. Genome-wide studies in the yeast *Saccharomyces cerevisiae* identified a set of genes associated with telomere length maintenance (*TLM* genes). Among the *tlm* mutants with short telomeres, we found a strong enrichment for those affecting vacuolar and endosomal traffic (particularly the endosomal sorting complex required for transport [ESCRT] pathway). Here, we present our results from investigating the surprising link between telomere shortening and the ESCRT machinery. Our data show that the whole ESCRT system is required to safeguard proper telomere length maintenance. We propose a model of impaired end resection resulting in too little telomeric overhang, such that Cdc13 binding is prevented, precluding either telomerase recruitment or telomeric overhang protection.

## IMPORTANCE

Telomeres are the ends of eukaryotic chromosomes. They are necessary for the proper replication of the genome and protect the chromosomes from degradation. In a large-scale systematic screen for mutants that affect telomere length in yeast, we found that mutations in any of the genes encoding the ESCRT complexes, required for the formation of transport vesicles within the cell, cause telomere shortening. We carried out an analysis of the mechanisms disrupted in these mutants and found that they are defective for the ability to elongate short telomeres, probably due to faulty end processing. We discuss the significance of these findings and how they could be relevant to anticancer therapies.

## INTRODUCTION

Telomeres preserve genome stability and promote cell viability by protecting the eukaryotic chromosomal ends from fusion, degradation, and unwanted double-strand break repair events. Telomeres consist of repetitive DNA elements and associated binding proteins, some of which are specific to telomere function (1). Telomeres end in a 3′ single-stranded DNA (ssDNA) overhang that is recognized by the Cdc13-Stn1-Ten1 (CST) complex, which participates in telomere elongation and protects telomeres from unwanted DNA double-strand repair mechanisms, such as exonuclease 1 (Exo1)-mediated resection (2). Due to the end replication problem, telomeres shorten gradually with each cell division, leading to replicative senescence or apoptosis upon reaching a critical minimal length (1). Yeast cells constitutively express telomerase, rendering *Saccharomyces cerevisiae* an excellent model organism to study telomerase regulation. Genome-wide studies of yeast mutant strains have revealed roughly 500 genes affecting telomere length (telomere length maintenance [TLM] genes) (3–7). The deletion of any TLM gene leads to either telomere shortening or lengthening. Among these genes, nine encode proteins of the endosomal sorting complex required for transport (ESCRT) system. Mutations in any of these nine genes result in telomeres that are shorter than those of the corresponding wild-type yeast. The ESCRT system consists of four multiprotein complexes (ESCRT-0, -I, -II, and -III) and associated proteins (8) that are involved in membrane deformation and scission events. In endosomal sorting, most data suggest sequential activity of the different subcomplexes to accomplish cargo protein recognition and sorting, as well as multivesicle body formation (9). In addition to endosomal sorting, certain ESCRT complexes are also involved in promoting cytokinesis, enveloped viral budding, autophagy, and nuclear envelope reformation (10–12). Furthermore, some ESCRT factors have also been associated with chromatin remodeling. The deletion of ESCRT proteins causes defects in chromosomal segregation in humans and in yeast, linking them to genomic stability and integrity (13).

We explored telomere function in the context of single *ESCRT* gene deletions to investigate the surprising link between telomere shortening and the ESCRT machinery. Our results show that the entire ESCRT system is required to safeguard proper telomere length maintenance. Based largely on our genetic studies, we propose a model of impaired telomere end resection in *ESCRT* gene deletion mutants (referred to hereinafter as *ΔESCRT* mutants), resulting in too little telomeric overhang such that telomerase recruitment/elongation becomes compromised.

## RESULTS

**FIG 1:**
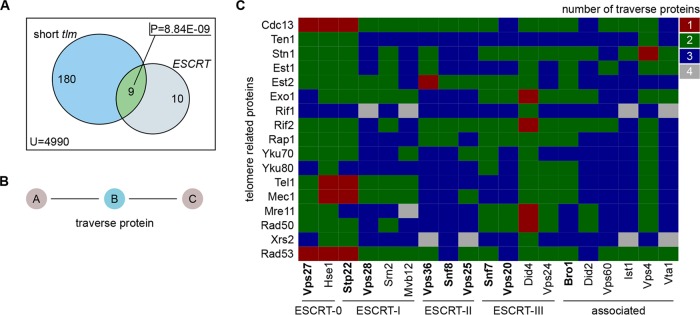
*ESCRT* genes are involved in telomere length maintenance. (A) Venn diagram depicting the overlap of *ESCRT* genes and short *tlm* mutants, whose *P* value (P) was calculated with a Fisher’s exact test. The set sizes are given per set, and the total number of genes that were considered for this test is stated as U (reference set size). (B) A traverse protein is defined as a protein that links two other proteins in the protein-protein interaction network. To reach protein C from A (or vice versa), protein B has to be traversed. (C) Matrix of shortest-path distances, given as the number of traverse proteins, between members of the ESCRT family (columns) and central telomere proteins (rows). Shortest-path investigation was done using the *igraph* library of program R. Boldface marks members of the ESCRT family whose corresponding mutants showed shortened telomeres.

Based on our findings that 9 of 19 *ESCRT* genes are significantly enriched within the subset of short *tlm* mutants (with a *P* value of 8.84E−09, Fisher’s exact test) (Fig. 1A), we examined the entire ESCRT family for its involvement in telomere length maintenance. To this end, we (i) analyzed protein-protein interaction (PPI) data and (ii) investigated the telomeric system in the context of single *ESCRT* gene deletions.

In order to gain insight into the connections of telomere-related proteins and the ESCRT family, we explored a manually curated *Saccharomyces cerevisiae* PPI network (5,592 proteins and 28,581 interactions). This network consisted exclusively of experimentally verified binary PPIs, rendering it a highly reliable interaction source (compiled analogously to the human interactome in Chapple et al. [14]). We defined a set of central telomere proteins (CTPs) (5, 6), consisting of all subunits of the CST (Cdc13, Stn1, and Ten1) capping complex, the Ku complex (Yku70 and Yku80), the MRX complex (Mre11, Rad50, and Xrs2), and the proteins Rif1, Rif2, Rap1, Exo1, Est1, Est2, Tel1, and Mec1. These proteins are part of the basic machinery involved in telomere length maintenance. We then carried out a shortest-path analysis, measuring the distances between ESCRT components and CTPs within the PPI network. Our results revealed that most ESCRT components were connected by at least two in-between (traverse) (Fig. 1B) proteins to CTP members (Fig. 1C). Remarkably, however, six ESCRT factors had a distance of only one traverse protein to a central telomere protein. Figure S1 in the supplemental material shows the induced subnetwork for all of those PPIs (31 proteins and 64 interactions). Interestingly, Cdc13 and the ESCRT-0 complex, especially Vps27, form a dense subnetwork whose traverse proteins are all protein kinases (see Fig. S1, orange). This analysis therefore suggests a strong association of ESCRT-0 to the telomeric system via Cdc13. We thus decided to utilize *VPS27* for a further in-depth investigation of the link between telomere shortening and the ESCRT machinery.

**FIG 2:**
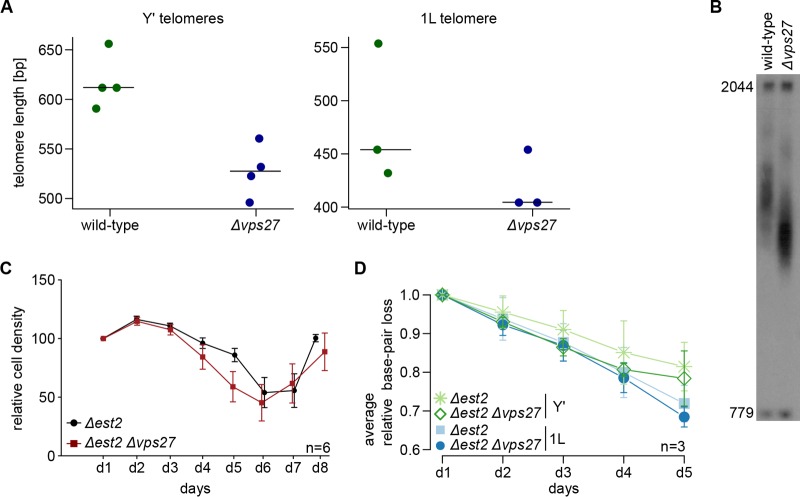
Telomerase-dependent telomere elongation is disturbed in *vps27* mutants. (A) Telomere length was measured by telomere PCR for wild-type and *Δvps27* cells after growth to at least 150 generations (six serial restreaks). Length was examined for six Y' telomeres and the 1L telomere. Horizontal bars represent the medians of the respective data points. (B) Representative Southern blot that could be used to measure Y' telomere lengths in wild-type and *Δvps27* cells. (C) Senescence curves of *Δest2* single and *Δest2 Δvps27* double mutants. For each mutant type, senescence was recorded for six different spore colonies (*n* = 6). Error bars represent the standard errors of the means. Two fragments, 2044 and 779 bp long, serve as size markers in telomere Southern blots. (D) Relative base pair loss of six Y' telomeres, as well as the 1L telomere, in *Δest2* single and *Δest2 Δvps27* double mutants. Days 1 to 5 correspond to the first five time points of the senescence curve in panel C. Relative base pair loss was calculated as the ratio of the number of base pairs on day *x* to the number of base pairs on day 1. The average relative base pair loss in three tetrads (*n* = 3) per day is shown. Error bars indicate means ± standard deviations. Figure S3 in the supplemental material shows the individual data averaged in panel D.

Initially, we measured telomere length after at least 150 generations (six serial restreaks) by telomere PCR (15) for different telomeres (Y'-containing telomeres and telomere 1L) in wild-type and *Δvps27* cells. Figure 2A shows the gel quantification for the telomere PCR, and Fig. 2B a representative Southern blot to measure telomere lengths for Y' telomeres only. The shortening of telomeres in *Δvps27* cells was striking, as previously reported (5, 16). As telomere length in budding yeast is primarily maintained by telomerase, we explored whether the loss of telomerase function affected the same pathway as that impaired in *Δvps27* cells. We deleted the *EST2* gene, encoding the catalytic subunit of telomerase, and recorded the growth potential of cells (relative cell density) and the corresponding telomere-shortening rate in cells with or without the deletion of the *VPS27* gene. Due to their lack of telomerase-dependent telomere elongation, *Δest2* mutants senesce after a certain number of passages; rare survivors appear later and take over the population. We found that the onset of senescence in *Δest2 Δvps27* double mutants was similar to that seen in the single *Δest2* mutants (Fig. 2C) and that the rates of telomere shortening were comparable in both mutants (Fig. 2D). We thus conclude that the telomere shortening in *Δvps27* cells is due to an effect on a telomerase-mediated mechanism.

**FIG 3:**
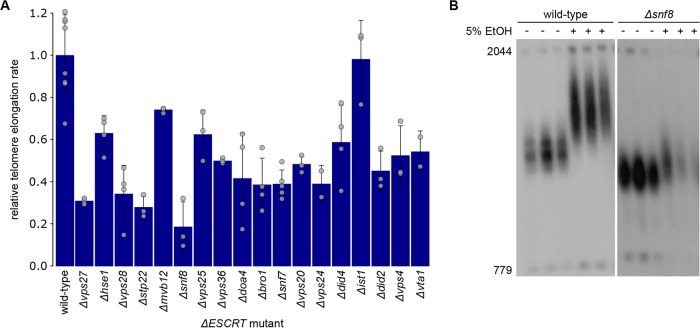
Decreased elongation rates of *ΔESCRT* mutants in response to ethanol stress. (A) Telomere length was measured by TelQuant (18). Standard deviations, as well as values for individual measurements (grey circles), are shown. The elongation rate was calculated as the difference between the final telomere length (after exposure to 5% ethanol over 60 generations) and the initial telomere length (before applying ethanol stress), divided by the initial length of each strain. Afterward, the elongation rate was normalized to the wild-type elongation rate, which was taken as one. (B) Southern blot showing wild-type and *Δsnf8* mutant telomere lengths before (−) and after (+) exposure to ethanol (EtOH) stress (5% ethanol in standard YP medium) of three independent cultures each.

In order to further confirm a defect in telomerase-mediated elongation in *ΔESCRT* mutants, we took advantage of previous work, which demonstrated that the exposure of cells to ethanol stress leads to increased telomere elongation via telomerase (17). We measured telomere length by Southern blot analysis in wild-type cells and *ΔESCRT* mutants exposed to ethanol (5% ethanol in liquid glucose-based YP medium) over 60 generations. The average telomere length was calculated using TelQuant (18). As expected, *ΔESCRT* mutants exhibited shorter telomeres than wild-type cells. Moreover, *ΔESCRT* mutants showed a weaker response to ethanol than the wild-type cells in terms of telomere elongation, with *Δvps27*, *Δstp22*, and *Δsnf8* mutants exhibiting the strongest effects (Fig. 3A and B; see also Fig. S2 in the supplemental material). This observation suggests that the entire ESCRT family is involved in telomerase-dependent elongation and that it is required for normal telomere maintenance, as well as for the physiological response to external signals.

**FIG 4:**
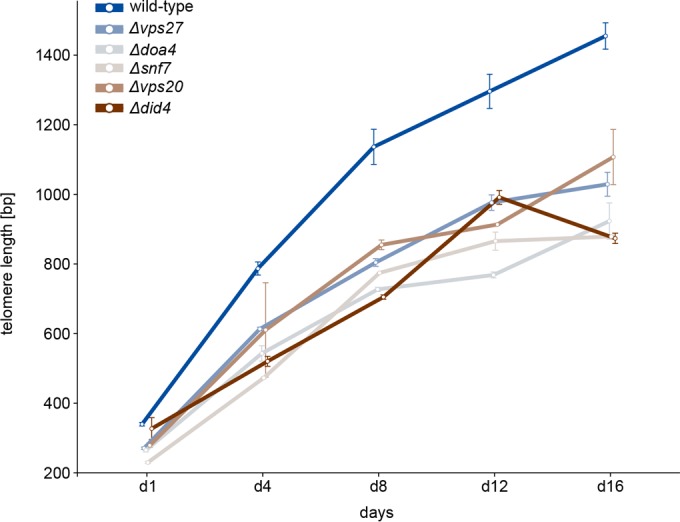
Reduced telomere elongation in *ΔESCRT* mutants despite forced telomerase recruitment. Kinetics of telomere elongation for mutants with the *ΔESCRT* mutations shown and the wild type. Strains carrying plasmids expressing the Cdc13-Est1 fusion protein (or an empty vector control) were streaked on –LEU plates for eight passages. On the indicated days, DNA was extracted, digested with *XhoI*, and analyzed by Southern blotting*.* Days 4, 8, 12, and 16 correspond to passages 2, 4, 6, and 8. Error bars indicate means ± standard deviations.

A bioinformatics analysis of gene expression in *ΔESCRT* deletion mutants did not reveal dramatic changes in the expression of telomere-related genes. Given the strong connectivity of Vps27 to Cdc13 in the protein-protein interaction network reported above, we investigated this link in further detail. Cdc13 is the major telomere ssDNA binding protein in yeast and regulates the access of telomerase to the chromosomal ends (19). Depending on the cell cycle stage, Cdc13 is bound to the telomere in different complexes. Either it is bound to the telomeric overhang as a member of the CST capping complex or it interacts with Est1 to promote telomerase recruitment (19). Est1 is a subunit of the telomerase ribonucleoprotein complex and is important for *in vivo* telomerase activity at telomeres (20). We forced telomerase to be tethered to the telomeric overhang by using a Cdc13-Est1 fusion protein, resulting in vigorous telomere elongation in wild-type cells. This fusion protein can be used to distinguish between defective telomerase recruitment and malfunctioning telomerase activation, although it does require the correct generation of a G overhang, which provides a Cdc13 binding site. If the absence of the ESCRT subunits impairs telomere elongation due to reduced telomerase recruitment, tethering telomerase to the telomere by expressing the Cdc13-Est1 fusion may bypass the problem and lead to wild-type telomere elongation. If, on the other hand, telomerase activity itself is impaired in the absence of ESCRT subunits, telomere elongation will be still reduced in the presence of the Cdc13-Est1 fusion protein. We expressed this fusion protein in six *ΔESCRT* mutants (*Δvps27*, *Δsnf8*, *Δvps20*, *Δsnf7*, *Δdid4*, and *Δdoa4* mutants) and evaluated the kinetics of telomere elongation. We observed that in comparison to that in wild-type cells, telomere elongation was reduced in all mutants, despite the “forced” recruitment (Fig. 4). These data suggest that in *ΔESCRT* mutants, either the activity of telomerase is compromised or the G overhang is not properly regulated, in turn preventing telomerase recruitment even when it is forced to be present at telomeres.

**FIG 5:**
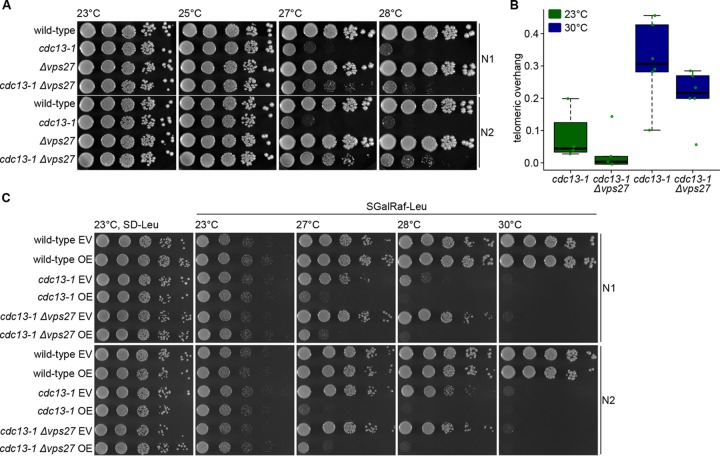
Deletion of *VPS27* rescues *cdc13-1* capping-defective telomeres. (A) Cells with the indicated genotypes were spotted in 10-fold serial dilutions onto standard YPD plates and incubated at different temperatures for 3 days. Two biological replicates are shown (N1 and N2). (B) Boxplot showing the amount of telomeric overhang for *cdc13-1* single and *cdc13-1 Δvps27* double mutants at permissive temperature (in green) and after heat-shock treatment (1 h at 30°C; in blue). Green dots indicate the individual data points. (C) As described for the experiment whose results are shown in panel A, the indicated mutants were spotted onto plates containing the indicated media. Cells contained either an Exo1 overexpression plasmid (OE) or the corresponding vector control (empty vector [EV]). Exo1 overexpression was induced by growth on galactose-containing plates. Plates were incubated at the indicated temperatures for 3 days. SD-Leu, yeast synthetic dropout medium without leucine (control); SGalRaf-Leu, SD-Leu medium containing 1% raffinose and 2% galactose.

We set out to test whether end resection and, hence, G tail formation is affected in *Δvps27* mutants. Using *cdc13-1*, a temperature-sensitive allele of *CDC13*, telomere uncapping can be induced by changing temperatures. After shifting *cdc13-1* cells to their nonpermissive temperature (>27°C), telomeres become uncapped and extensively long G overhangs are generated in a nuclease-dependent manner, resulting in a checkpoint-mediated cell cycle arrest. Indeed, when nucleases like Exo1 are deleted in *cdc13-1* cells, the cells are viable even at temperatures above 27°C. We examined *cdc13-1 Δvps27* double mutants for their viability at the permissive (23°C and 25°C) and nonpermissive (27°C and 28°C) temperatures. Deleting *VPS27* allowed the growth of *cdc13-1* cells at elevated temperatures (Fig. 5A). This finding is in agreement with the results of Addinall et al., who reported that a deletion of *HSE1* (a Vps27 interactor) suppresses the temperature sensitivity of *cdc13-1* (21). Hence, the deletion of ESCRT-0 seems to protect the cells from uncapped telomeres. This finding holds true for almost all ESCRT gene deletions (see Fig. S3 in the supplemental material).

Both reduced Exo1-mediated resection and/or defective cell cycle checkpoint activation could potentially account for the increased growth of *cdc13-1* cells at elevated temperature. First, we explored whether the deletion of *VPS27* influences Exo1-mediated resection. Degradation of the 5′ end of uncapped telomeres leads to increased amounts of 3′ telomeric single-stranded overhang. We measured the amount of telomeric ssDNA in *cdc13-1* and *cdc13-1 Δvps27* cells at 23°C or after 1 h of incubation at 30°C to induce telomere uncapping. As expected, we saw elevated levels of 3′ ssDNA in *cdc13-1* cells at high temperature using a dot blot approach under native and denaturing conditions (Fig. 5B; see also Fig. S4 in the supplemental material). The amount of 3′ telomeric overhang was decreased in the double mutant at both temperatures (Fig. 5B). This observation suggests a compromised Exo1-mediated resection in *cdc13-1 Δvps27* double mutants. In addition, overexpression of Exo1 in *cdc13-1 Δvps27* cells (but not an empty vector) abolished the rescue effect (Fig. 5C), supporting the idea of a defective Exo1-mediated resection upon *VPS27* deletion. In order to test whether *Δvps27* mutants may have lower expression of *EXO1*, we assessed the protein levels of Exo1 in *Δvps27* single mutants and *cdc13-1 Δvps27* double mutants compared to the levels in wild-type cells after the induction of telomere uncapping by transfer to high temperature (1 h at 30°C). We also examined the degradation kinetics of Exo1 in the double mutants using cycloheximide chase experiments. The Exo1 levels were not altered by the deletion of *VPS27*, and there were no apparent differences in Exo1 protein degradation kinetics between *cdc13-1* and *cdc13-1 Δvps27* mutants. Together, these results suggest that the rescue of *cdc13-1* cells by the deletion of *ESCRT* components may be due to impaired resection of the telomere, although differences in *EXO1* expression cannot be held accountable.

**FIG 6:**
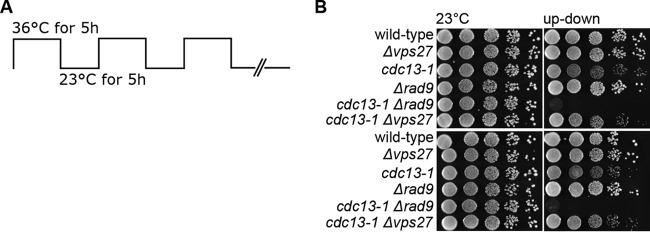
cdc13-1 *Δvps27* double mutants are viable in a up-down assay. (A) Schematic of the up-down assay employed. Cells were incubated at permissive temperature (23°C) for 5 h, followed by a phase of nonpermissive temperature (36°C) for 5 h. After three cycles, plates were kept on hold at 23°C. (B) Cells were spotted in 10-fold serial dilutions onto standard YPD plates that were incubated at oscillating temperatures for 3 days. Two biological replicates were spotted. Controls were incubated at 23°C.

Other than a defective Exo1-mediated resection, improper DNA damage checkpoint activation could also explain the apparent rescue of *cdc13-1* capping-defective telomeres. An up-down assay (Fig. 6A) was previously developed in which cells are subjected to cycles of high and low temperature (21). As the temperature sensitivity of *cdc13-1* mutants is reversible, the cells can maintain viability upon short exposures to the nonpermissive temperature if returned to permissive temperature afterward. Mutants defective for the DNA damage checkpoint activation fail to arrest in response to DNA damage, and recovery is not possible (21). Under these conditions, mutations in *EXO1* suppress *cdc13-1* defects, whereas a deletion of the *RAD9* checkpoint gene is unable to do so (21). The results in Fig. 6B show that the *cdc13-1 Δvps27* mutants were viable in this assay, in contrast to checkpoint-defective *Δrad9* cells, indicating that checkpoint activation is normal in the absence of *VPS27*.

## DISCUSSION

Based on an enrichment of *ESCRT* genes within the subset of short *tlm* mutants (Fig. 1A), we explored the possible role of ESCRT factors in telomere maintenance. We found that telomere shortening is a general phenomenon for *ΔESCRT* mutants, indicating a general requirement of a fully functional ESCRT system for proper telomere length homeostasis. Our in-depth analysis of *Δvps27* cells points to a model of diminished resection of the telomeric ends, resulting in short 3′ overhangs; under these conditions, telomerase cannot be normally recruited or activated. As a result, telomeres are short and are not extended normally under conditions of telomerase activation (such as the addition of ethanol or telomerase tethering). On one hand, this inefficiency may be beneficial for dysfunctional telomeres, which become susceptible to end resection, but on the other hand, it impairs telomere extension by telomerase and could prevent efficient homologous recombination at double-stranded DNA (dsDNA) breaks elsewhere in the genome. The idea of hampered Exo1-mediated resection resulting in decreased amounts of telomeric overhang is supported by several findings: (i) the deletion of any *ESCRT* gene rescued the temperature sensitivity of *cdc13-1* mutants (Fig. 5A; see also Fig. S3 in the supplemental material), (ii) after telomere uncapping at high temperature, *cdc13-1 Δvps27* double mutants had less telomeric overhang than *cdc13-1* single mutants (Fig. 5B), and (iii) the overexpression of Exo1 abolished the rescue effect (Fig. 5C). In addition, resection-mediated generation of a G overhang is also required during the regular replication of eroded telomeres to provide a platform for telomerase-dependent extension. Defective telomerase-mediated telomere elongation is supported by our observations that (i) telomeres shorten in all *ΔESCRT* mutants under standard conditions and fail to elongate in the presence of ethanol stress (Fig. 3; see also Fig. S2), (ii) similar senescence rates are observed in *Δest2 Δvps27* double and *Δest2* single mutants (Fig. 2C), and (iii) there is reduced telomere elongation in all *ΔESCRT* mutants despite forced telomerase recruitment (Cdc13-Est1 fusion protein) (Fig. 4). The ESCRT system plays a central role in the transport of proteins within the cells; our results are consistent with a role of this pathway in the transport of a factor(s) involved in telomere overhang processing. Further experiments will be aimed at identifying these factors.

The results from our PPI network analysis leave room to speculate that protein kinases interlink Cdc13 and Vps27 and serve in a feedback loop to regulate telomere homoeostasis via the ESCRT machinery, whereby Vps27 represents the entry point to it. Indeed, the recruitment of the ESCRT machinery depends on the phosphorylation of Vps27 (22).

Since 85 to 90% of human primary tumors show telomerase upregulation, ESCRT factors could serve as new targets to hinder telomerase function in tumor cells. However, as ESCRT factors are essential to mammals and disruption of them bears the potential for oncogenicity, thorough verification of the proposed mechanism in mammalian systems has to be undertaken. In general, ESCRT-0 seems most suitable as an anticancer drug target compared to other mammalian ESCRT complexes, such as ESCRT-III, which is sufficient to achieve minimal ESCRT function. This later subcomplex has been shown to be involved in crucial nuclear functions (chromatin remodeling, chromosomal segregation, and nuclear envelope reformation) (10, 23). As it has evolved rather recently, ESCRT-0 might be more easily substitutable and its dysfunction might not cause side effects as severe as in other ESCRT disruptions. Data from Toyoshima and colleagues further strengthen the idea of the suitability of ESCRT-0 as an anticancer drug target. They have shown that Hgs (the mammalian homologue of Vps27) is upregulated in several tumors (e.g., melanoma, cervix, and liver tumors) and that its tumorigenic potential could be reduced by small interfering RNA (siRNA)-facilitated downregulation (24).

## MATERIALS AND METHODS

### *In silico* analyses.

All *in silico* analyses were based on the list of previously found telomere length maintenance (TLM) genes and performed using the R environment. The list of *ESCRT* genes is presented in Table S3 in the supplemental material, and that of the *TLM* genes in Table S4. The *TLM* gene list was compiled and cleaned of duplicates and identifier (ID) problems using R. If required, ID mappings were done with the Bioconductor package for yeast annotations (*org.Sc.sgd.db*). The *TLM* gene list finally comprised 482 genes, of which 286 show short telomeres when mutated and 182 show long telomeres.

### (i) Enrichment analyses.

For testing the significance of the overlap of two gene sets, the default two-sided Fisher’s exact test functionality from the R program was employed. The reference (background) set contained 4,990 genes in total.

### (ii) Protein-protein interaction network.

The manually curated *Saccharomyces cerevisiae* interaction network was kindly provided by Christine Brun. It was compiled analogously to the human protein interaction network prepared by Chapple and colleagues (14). Shortest-path investigation was performed using the *igraph* library within R. Network visualization was done in Cytoscape (version 3.2.1).

### (iii) Knockout gene expression data.

A submatrix of *M* values composed of *Δescrt*-only columns and CTP rows was extracted from the genome-wide mRNA expression data set released by the Holstege laboratory in April 2014 (25). Only deletion strains classified as responsive (different from the wild type) by the authors were considered (fold change of >1.7 and *P* value of <0.05). Twelve of the 19 ESCRT factor-encoding genes were contained within the samples of this data. Data preprocessing and visualization were done using R and the *corrplot* package.

### Experimental analyses. (i) Yeast strains.

The yeast strains used in this study are listed in Table S1 in the supplemental material. Haploid strains were derived from the listed diploids. Unless specified differently, cultures were grown in standard yeast complete medium (yeast extract-peptone-dextrose [YPD]).

### (ii) Telomere length measurements.

Telomere length was measured either by Southern blotting as previously described (6, 18) or by telomere PCR as described below.

The indicated strains were serially restreaked six times on YPD plates at 30°C. Cell pellets of overnight cultures from the last restreak were used for measuring telomere length by telomere PCR. One hundred fifty nanograms of genomic DNA in 10 µl of 1× NEBuffer 4 was denatured at 96°C for 10 min and cooled down to 4°C. For the C-tailing reaction, 1 µl tailing mix (0.2 µl terminal transferase at 20 U/µl, 0.1 µl 10× NEBuffer 4, 0.1 µl 10 mM dCTPs, 0.6 µl MilliQ water) was added and samples were incubated at 37°C for 30 min, 65°C for 10 min, and 96°C for 5 min. After cooling to 65°C, PCR cycling was initiated. Thirty microliters of preheated PCR mixture was added. Per reaction mixture volume, the PCR mixture contained 0.5 µl Q5 high-fidelity (HF) or Phusion Hot Start DNA polymerase, 0.3 µl oligo(dG) reverse primer (oBL359) (see Table S3 in the supplemental material), 0.3 µl telomere-specific forward primer (6Y′, oBL361, or 1L, oBL358) (see Table S2), 4 µl 2 mM deoxynucleoside triphosphates (dNTPs), 4 µl 10× PCR buffer, and 21 µl MilliQ water. The PCR cycling conditions were as follows: 3 min at 95°C, followed by 45 cycles of 95°C for 30 s, 63°C for 15 s, and 68°C for 20 s, and after an additional 5 min at 68°C, cooling to 4°C for holding.

### (iii) Senescence curves and rates of telomere shortening.

Spore colonies derived from diploid dissection were dissolved in 500 µl of water. After dilution in YPD medium to an optical density at 600 nm (OD_600_) of 0.01 (final concentration), cultures were incubated at 30°C for 24 h. Using a spectrophotometer, cell density was measured and cells were rediluted in 5 ml of YPD medium to an OD_600_ of 0.01 (final concentration). Cultures were rediluted and incubated in this way for 8 days. Every 24 h, cell samples were taken, pelleted, and stored at −20°C.

### (iv) Viability spotting experiments.

Unless specified differently, cells were 1:10 serially diluted in water, spotted onto standard YPD plates, and incubated at 30°C. The initial cell concentration was at an OD_600_ of 0.5. Images were taken after 2 to 3 days of growth.

The up-down protocol was adapted from Addinall et al. (21). Temperature oscillation was performed in a preheated (23°C) programmable Sanyo incubator. Controls were incubated at 23°C.

### (v) Measurements of ssDNA telomere overhang by dot blot assay.

Genomic DNA (gDNA) was purified using the Gentra Puragene yeast/bacteria kit (Qiagen GmbH). Isolated DNA were kept on ice for native conditions or denatured (1 µg gDNA, 100 µl 0.2 M NaOH) for 15 min at 65°C. Additionally, 8 µg gDNA was digested by *Escherichia coli* exonuclease 1 (2 µl Exo1 [NEB], 5 µl Exo1 buffer, MilliQ water to 50-µl total volume) for 2 h at 37°C. After dilution in 2× SSC (1× SSC is 0.15 M NaCl plus 0.015 M sodium citrate) to a final volume of 400 µl, DNA samples were spotted onto a positively charged nylon membrane (Amersham Hybond-N+) using a Bio-Dot microfiltration apparatus (Bio-Rad Laboratories GmbH). The membrane was washed thrice in tetramethylammonium chloride (TMAC) and once in 2× SSC (1 min per wash). Subsequent to cross-linking (Stratagene Stratalinker UV crosslinker 2400), the membrane was incubated at 47.5°C overnight with a digoxigenin (DIG)-labeled probe specific to the telomeric repeats (oBL207 in hybridization solution) (see Table S2 in the supplemental material). After washing and exposure, signal quantification was done using the Dot Blot Analyzer tool for ImageJ. The amount of telomeric overhang was calculated as follows: (native DNA − Exo1_bact_-digested DNA)/denatured DNA.

### (vi) Measurements of protein levels by Western blotting.

Telomere uncapping was induced by heat shock as described for the measurement of telomeric ssDNA overhang. The protein levels of exonuclease 1 were measured using a tandem affinity purification (TAP)-tagged Exo1. Western blots were performed as previously described (26).

For the protein degradation kinetics experiment, different samples of the same cultures were taken at different time points. Overnight cultures were diluted to an OD_600_ of 0.2 and grown to an OD_600_ of 0.5 at 23°C. Before shifting the cultures to 30°C for 1 h, the control sample was taken. Cycloheximide (CHX; Sigma Aldrich Co. LLC) was added to the cultures to a final concentration of 200 µg/ml after samples for heat-shocked controls were taken (denoted as time point zero). At 30, 45, 60, and 75 min after the addition of CHX, samples were taken. The antibodies used in this experiment were peroxidase anti-peroxidase (PAP) soluble complex antibody (product number P1291; Sigma Aldrich Co. LLC) at 1:3,000, Pgk1 (gift from the Knop laboratory) at 1:25,000, anti-rabbit IRDye 800CW antibody (product number 926-32211; LI-COR Biosciences GmbH), and anti-mouse IRDye 680RD antibody (product number 926-68070; LI-COR Biosciences GmbH), both at 1:10,000. Image acquisition was done on an Odyssey (LI-COR Biosciences GmbH) system. Bands were quantified using Image Studio (version 4.0).

## SUPPLEMENTAL MATERIAL

Figure S1  Protein kinases interlink Cdc13 with ESCRT-0. Subnetwork of all central telomere proteins and ESCRT factors that are connected in the protein-protein interaction network by one traverse protein (see Fig. 1C). The symbols correspond to the encoding genes. Download Figure S1, PDF file, 0.3 MB

Figure S2  Telomere lengths of *ΔESCRT* mutants in response to 5% ethanol stress over 60 generations. (A to D) Wild-type and *ΔESCRT* mutants were grown for 60 generations in liquid YPD or in YPD with 5% ethanol. DNA was extracted after 60 generations, digested with XhoI, and analyzed by Southern blotting. The membrane was probed with a telomere sequence and with unique genomic sequences used as markers (779 bp and 2044 bp) to enable telomere length measurements. Download Figure S2, PDF file, 0.2 MB

Figure S3  All *ΔESCRT* mutants rescue *cdc13-1* capping-defective telomeres. The indicated strains were spotted in 10-fold serial dilutions onto standard YPD plates and incubated at permissive (23°C and 25°C) and nonpermissive (27°C and 28°C) temperatures for 3 days. Each *cdc13-1 ΔESCRT* double mutant and its respective single mutants were spotted in biological duplicates. (A) ESCRT-0, (B) ESCRT-I, (C) ESCRT-II, (D) ESCRT-III, (E) ESCRT-III a, and (F) ESCRT associated. (G) Plot of mean fitness values from quantitative fitness analysis (QFA) using *cdc13-1* as query background and URA3 as control background. The plot was generated using QFA data and the visualization tool DIXY (http://bsu-srv.ncl.ac.uk/dixy/viz/) from Addinall et al. (doi:10.1371/journal.pgen.1001362). Download Figure S3, PDF file, 0.7 MB

Figure S4  Decreased amounts of telomeric overhang in *cdc13-1 Δvps27* double mutants. The amounts of 3′ telomeric ssDNA in *cdc13-1* single and *cdc13-1 Δvps27* double mutants were quantified using a dot blot assay. Samples were taken after growth at permissive temperature (23°C) and after heat shock at 30°C for 1 h. Subsequent to cross-linking, the membrane was incubated overnight with a DIG-labeled probe specific to the telomeric repeats (oBL207, CACCACACCCACACACCACACCCACA). (A) Image of the membrane onto which two technical (rows) and six biological (columns) replicates (*n_i_*) were spotted. DNA samples of native, denatured, and bacterial-Exo1 (Exo1_bact_)-digested DNA were spotted. Signal quantification was done using the Dot Blot Analyzer tool for ImageJ. The amount of telomeric overhang (Fig. 5B) was calculated as (native DNA – Exo1_bact_-digested DNA)/denatured DNA. (B) Model of the membrane visualized as extrapolated pixel intensities by the Dot Blot Analyzer tool for ImageJ. Download Figure S4, PDF file, 0.2 MB

Table S1  Yeast strains used in this study. Table S1, PDF file, 0.5 MB

Table S2  Oligonucleotides used in this study. Table S2, PDF file, 0.4 MB

Table S3  List of all ESCRT genes. Table S3, PDF file, 0.3 MB

Table S4  List of all TLM genes denoted by open reading frame identifiers (ORFs). Table S4, PDF file, 0.5 MB
