# Supplementary figures and images for: Role of the ESCRT Complexes in Telomere Biology
Source: mBio. 2016 Nov 8;7(6):e01793-16. doi: 10.1128/mBio.01793-16 (PMC5101353; doi:10.1128/mBio.01793-16)

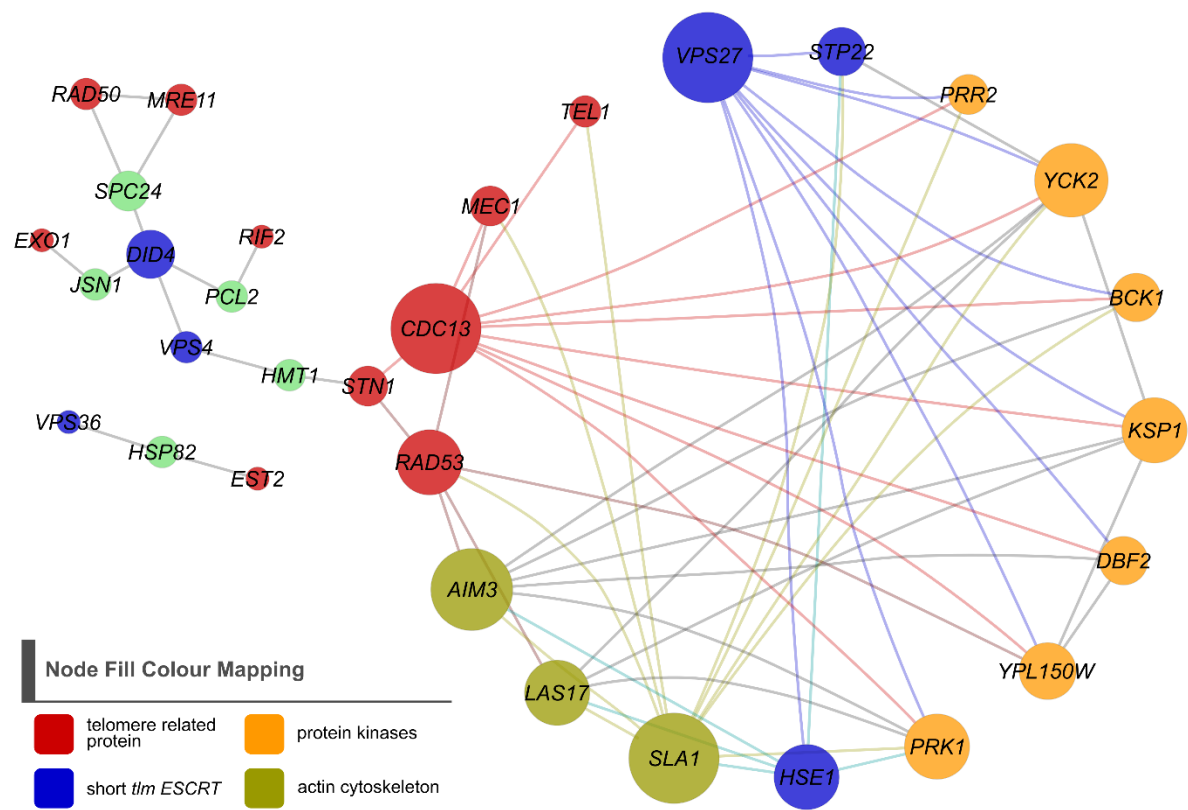

Suppl. Fig. 1

Supplement: Figure S1 — Protein kinases interlink Cdc13 with ESCRT-0. Subnetwork of all central telomere proteins and ESCRT factors that are connected in the protein-protein interaction network by one traverse protein (see Fig. 1C). The symbols correspond to the encoding genes. Download [file mbo006163063sf1.pdf]

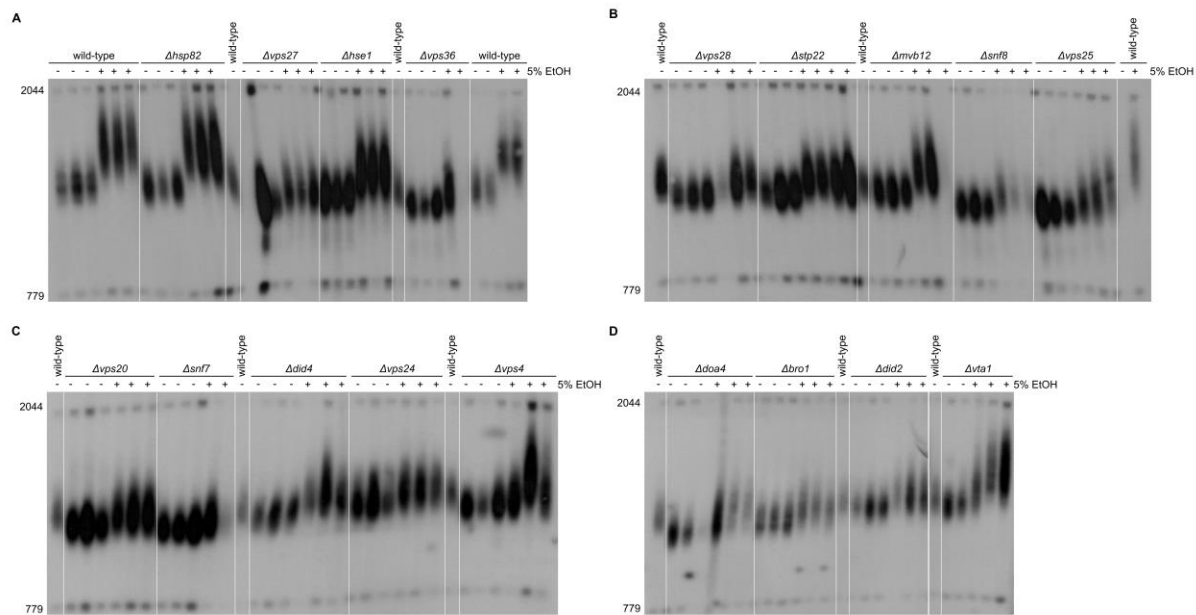

Suppl. Figure 2

Supplement: Figure S2 — Telomere lengths of ΔESCRT mutants in response to 5% ethanol stress over 60 generations. (A to D) Wild-type and ΔESCRT mutants were grown for 60 generations in liquid YPD or in YPD with 5% ethanol. DNA was extracted after 60 generations, digested with XhoI, and analyzed by Southern blotting. The membrane was probed with a telomere sequence and with unique genomic sequences used as markers (779 bp and 2044 bp) to enable telomere length measurements. Download [file mbo006163063sf2.pdf]

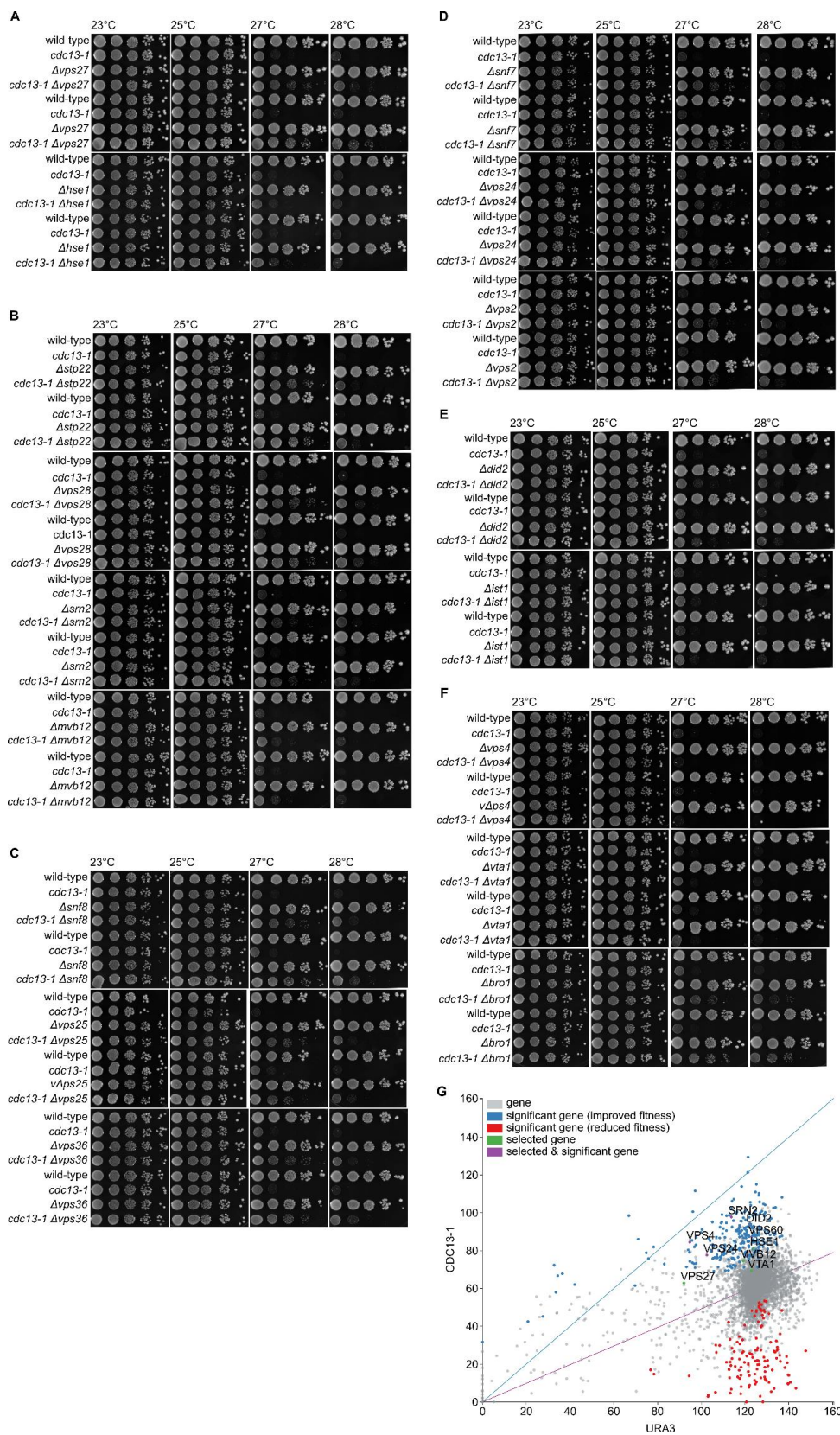

Figure S3

Supplement: Figure S3 — All ΔESCRT mutants rescue cdc13-1 capping-defective telomeres. The indicated strains were spotted in 10-fold serial dilutions onto standard YPD plates and incubated at permissive (23°C and 25°C) and nonpermissive (27°C and 28°C) temperatures for 3 days. Each cdc13-1 ΔESCRT double mutant and its respective single mutants were spotted in biological duplicates. (A) ESCRT-0, (B) ESCRT-I, (C) ESCRT-II, (D) ESCRT-III, (E) ESCRT-III a, and (F) ESCRT associated. (G) Plot of mean fitness values from quantitative fitness analysis (QFA) using cdc13-1 as query background and URA3 as control background. The plot was generated using QFA data and the visualization tool DIXY (http://bsu-srv.ncl.ac.uk/dixy/viz/) from Addinall et al. (doi:10.1371/journal.pgen.1001362). Download [file mbo006163063sf3.pdf]

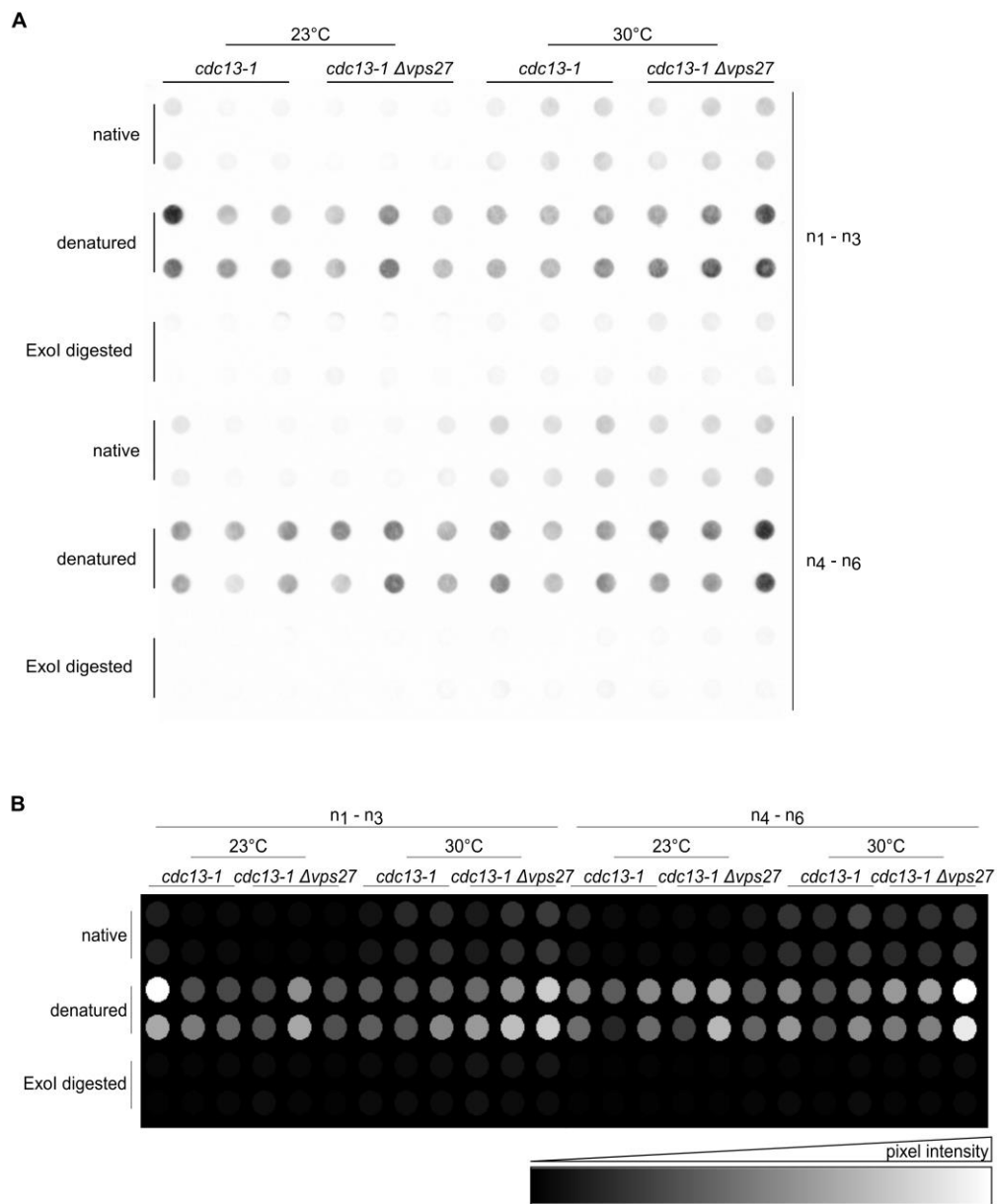

Figure S4

Supplement: Figure S4 — Decreased amounts of telomeric overhang in cdc13-1 Δvps27 double mutants. The amounts of 3′ telomeric ssDNA in cdc13-1 single and cdc13-1 Δvps27 double mutants were quantified using a dot blot assay. Samples were taken after growth at permissive temperature (23°C) and after heat shock at 30°C for 1 h. Subsequent to cross-linking, the membrane was incubated overnight with a DIG-labeled probe specific to the telomeric repeats (oBL207, CACCACACCCACACACCACACCCACA). (A) Image of the membrane onto which two technical (rows) and six biological (columns) replicates (ni) were spotted. DNA samples of native, denatured, and bacterial-Exo1 (Exo1bact)-digested DNA were spotted. Signal quantification was done using the Dot Blot Analyzer tool for ImageJ. The amount of telomeric overhang (Fig. 5B) was calculated as (native DNA – Exo1bact-digested DNA)/denatured DNA. (B) Model of the membrane visualized as extrapolated pixel intensities by the Dot Blot Analyzer tool for ImageJ. Download [file mbo006163063sf4.pdf]
