# Supplementary material for: Role of the ESCRT Complexes in Telomere Biology
Source: mBio. 2016 Nov 8;7(6):e01793-16. doi: 10.1128/mBio.01793-16 (PMC5101353; doi:10.1128/mBio.01793-16)
Supplement: Table S1 — Yeast strains used in this study. [file mbo006163063st1.pdf]

Suppl. Table 1. Yeast strains used in this study. Yeast strains used in this study were either derivatives from the BY4741 background (*his3Δ1*, *leu2Δ0*, *ura3Δ0*, *met15Δ0*) or from YKM139 (yAM344, yAM436, and yAM437; background *hmlΔ::ADE1 hmrΔ::ADE1 ade1-100 leu2-3,112 lys5 trp1::hisG ade3::GAL-HO*). Haploid strains were derived from the listed diploids.

**Code**	**Genotype**
yAM223	MATa *Δvps27::KAN*
yAM225	MATa/MATalpha *EST2/est2::HIS VPS27/vps27::KAN*
yAM229	MATa *CDC13::cdc13-1 HIS Δvps27::KAN*
yAM234	MATalpha *CDC13::cdc13-1 HIS Δvps27::KAN*
yAM235	MATa EXO1-TAP (HIS) *Δvps27::KAN*
yAM269	MATa/MATalpha *HSC82/hsc82::KAN CDC13/CDC13::cdc13-1 HIS*
yAM270	MATa/MATalpha CDC13-TAP (HIS) *VPS27/vps27::KAN*
yAM274	MATa *Δstp22::KAN*
yAM275	MATa *Δvps28::KAN*
yAM278	MATa *Δsnf8::KAN*
yAM282	MATa *Δsnf7::KAN*
yAM285	MATa *Δdid2::KAN*
yAM290	MATa *Δbro1::KAN*
yAM297	MATalpha *Δvps27::NAT*
yAM325	MATa/MATalpha SAE2-TAP (HIS) *VPS27/vps27::KAN*
yAM386	MATa/MATalpha *CDC13/CDC13::cdc13-1 KAN VPS27/vps27::NAT* EXO1-TAP (HIS)
yAM387	MATa *CDC13::cdc13-1 KAN Δvps27::NAT* EXO1-TAP (HIS)
yAM433	MATa *Δvps27::KAN*
yAM436	MATa *Δvps27::KAN*
yAM437	MATa *Δvps27::KAN*
yAM443	MATa/MATalpha *CDC13/CDC13::cdc13-1 HIS HSE1/hse1::KAN*
yAM444	MATa/MATalpha *CDC13/CDC13::cdc13-1 HIS STP22/stp22::KAN*
yAM445	MATa/MATalpha *CDC13/CDC13::cdc13-1 HIS VPS28/vps28::KAN*
yAM446	MATa/MATalpha *CDC13/CDC13::cdc13-1 HIS SRN2/srn2::KAN*
yAM447	MATa/MATalpha *CDC13/CDC13::cdc13-1 HIS VPS4/vps4::KAN*
yAM448	MATa/MATalpha *CDC13/CDC13::cdc13-1 HIS VTA1/vta1::KAN*
yAM449	MATa/MATalpha *CDC13/CDC13::cdc13-1 HIS MVB12/mvb12::KAN*
yAM450	MATa/MATalpha *CDC13/CDC13::cdc13-1 HIS SNF8/snf8::KAN*
yAM451	MATa/MATalpha *CDC13/CDC13::cdc13-1 HIS VPS25/vps25::KAN*

yAM452	MATa/MATalpha *CDC13/CDC13::cdc13-1 HIS VPS36/vps36::KAN*
yAM453	MATa/MATalpha *CDC13/CDC13::cdc13-1 HIS VPS20/vps20::KAN*
yAM454	MATa/MATalpha *CDC13/CDC13::cdc13-1 HIS SNF7/snf7::KAN*
yAM455	MATa/MATalpha *CDC13/CDC13::cdc13-1 HIS VPS24/vps24::KAN*
yAM456	MATa/MATalpha *CDC13/CDC13::cdc13-1 HIS VPS2/vps2::KAN*
yAM457	MATa/MATalpha *CDC13/CDC13::cdc13-1 HIS DID2/did2::KAN*
yAM458	MATa/MATalpha *CDC13/CDC13::cdc13-1 HIS VPS60/vps60::KAN*
yAM459	MATa/MATalpha *CDC13/CDC13::cdc13-1 HIS IST1/ist1::KAN*
yAM460	MATa/MATalpha *CDC13/CDC13::cdc13-1 HIS BRO1/bro1::KAN*
yBL7	MATa
yBL679	MATalpha
yJK162	MATa EXO1-TAP (HIS)
yJK199	MATalpha *CDC13::cdc13-1 HIS*
yJK244	MATa *CDC13::cdc13-1 KAN* EXO1-TAP (HIS)
ySLG60	MATalpha *Δrad9::KAN*
ySLG70	MATalpha *CDC13::cdc13-1 KAN Δrad9::KAN*
