# Supplementary material for: Role of the ESCRT Complexes in Telomere Biology
Source: mBio. 2016 Nov 8;7(6):e01793-16. doi: 10.1128/mBio.01793-16 (PMC5101353; doi:10.1128/mBio.01793-16)
Supplement: Table S2 — Oligonucleotides used in this study. [file mbo006163063st2.pdf]

**Table S2. Oligonucleotides used in this study.**

| Code | Name | Sequence |
|---|---|---|
| oBL29 | KAN/NAT reverse | CTGCAGCGAGGAGCCGTAAT |
| oBL207 | telomeric repeat | CACCACACCCACACACCACACCCACA |
| oBL358 | 1L forward | GCGGTACCAGGGTTAGATTAGGGCTG |
| oBL359 | oligo dG | $CGGGATCCG_{18}$ |
| oBL361 | 6Y' forward | TTAGGGCTATGTAGAAGTGCTG |
| oJK73 | *VPS27* forward | AGTTAGCACGCGCATATCCA |
| oJK74 | *VPS27* reverse | AGAACAAGAGCTGGACGGTG |
| oJK75 | *VPS27* forward | CCTGTGGTATGTTTTGAAGGGC |
