# Supplementary material for: Role of the ESCRT Complexes in Telomere Biology
Source: mBio. 2016 Nov 8;7(6):e01793-16. doi: 10.1128/mBio.01793-16 (PMC5101353; doi:10.1128/mBio.01793-16)
Supplement: Table S3 — List of all ESCRT genes. [file mbo006163063st3.pdf]

Suppl. Table 3. List of all ESCRT genes. The individual ESCRT subunits, associated proteins and their biological role. Alternative metazoan names are provided in brackets. Bold letters mark those members of the ESCRT family whose corresponding mutants showed short telomeres. MVB, MVB biogenesis; A, autophagy; V, viral budding; and C, cytokinesis.

Complex	Yeast	Metazoan	Biological Role			
			MVB	A	V	C
ESCRT-0	**Vps27**	HRS (HGS)	x	x		
	Hse1	STAM1,2	x	x		
ESCRT-I	**Stp22**	TSG101	x	x	x	x
	**Vps28**	VPS28	x	x	x	x
	Srn2	VPS37A-D	x	x	x	x
	Mvb12	MVB12A,B	x	x	x	x
ESCRT-II	**Snf8**	VPS22 (EAP30)	x	x		
	**Vps25**	VPS25 (EAP20)	x	x		
	**Vps36**	VPS36 (EAP45)	x	x		
ESCRT-III	**Vps20**	VPS20 (CHMP6)	x	x		
	Did4	VPS2A,B (CHMP2A,B)	x	x	x	x
	Vps24	VPS24 (CHMP3)	x	x	x	x
	**Snf7**	SNF7A–C (CHMP4A–C)	x	x	x	x
ESCRT-IIIa	Did2	DID2A,B (CHMP1A,B)	x		x	x
	Vps60	CHMP5	x			x
	Ist1	IST1	x	x	x	x
Other	Vps4	VPS4A,B	x	x	x	x
	Vta1	VTA1 (LIP5)	x	x	x	x
	**Bro1**	ALIX (AIP1)	x	x	x	x
