# Supplementary material for: Role of the ESCRT Complexes in Telomere Biology
Source: mBio. 2016 Nov 8;7(6):e01793-16. doi: 10.1128/mBio.01793-16 (PMC5101353; doi:10.1128/mBio.01793-16)
Supplement: Table S4 — List of all TLM genes denoted by open reading frame identifiers (ORFs). [file mbo006163063st4.pdf]

Suppl. Table 4. List of all *TLM* genes denoted by Open Reading Frame identifiers (ORFs). Asterisks mark those *TLM* genes that were detected by screening the non-essential deletion strain collections. Short *TLM* genes marked with an asterisk were used only to estimate the significance of the overlap of short *TLM* and *ESCRT* genes (Figure 1A).

TLM Phenotype	Open Reading Frame Identifier
**Short** (286)	YAL013W*, YAL016W*, YAL044C*, YAL056W*, YAR002W*, YAR003W*, YBL046W*, YBL058W*, YBL088C*, YBR035C*, YBR097W*, YBR103W*, YBR106W*, YBR112C*, YBR132C*, YBR175W*, YBR278W*, YBR279W*, YBR286W*, YCL008C*, YCL016C*, YCL061C*, YDL006W*, YDL020C*, YDL047W*, YDL074C*, YDL077C*, YDL081C*, YDL101C*, YDL118W*, YDL119C*, YDL185W*, YDL192W*, YDR027C*, YDR069C*, YDR080W*, YDR115W*, YDR121W*, YDR159W*, YDR206W*, YDR227W*, YDR295C*, YDR300C*, YDR310C*, YDR320C*, YDR363W-A*, YDR369C*, YDR418W*, YDR495C*, YDR532C*, YEL029C*, YEL033W*, YEL057C*, YER059W*, YER068W*, YER070W*, YFR040W*, YFR041C*, YGL024W*, YGL025C*, YGL058W*, YGL066W*, YGL070C*, YGL084C*, YGL127C*, YGL135W*, YGL167C*, YGL168W*, YGL173C*, YGL212W*, YGL244W*, YGR036C*, YGR042W*, YGR057C*, YGR072W*, YGR101W*, YGR104C*, YGR163W*, YGR229C*, YGR262C*, YGR270W*, YHL011C*, YHL020C*, YHR041C*, YHR059W*, YHR077C*, YHR081W*, YHR139C*, YHR167W*, YHR191C*, YIL009C-A*, YIL036W*, YIL042C*, YIL052C*, YIL077C*, YJL029C*, YJL071W*, YJL129C*, YJL140W*, YJL184W*, YJR102C*, YJR105W*, YKL009W*, YKL139W*, YKL168C*, YKL170W*, YKL204W*, YLL006W*, YLL027W*, YLR015W*, YLR025W*, YLR090W*, YLR148W*, YLR192C*, YLR233C*, YLR234W*, YLR240W*, YLR242C*, YLR318W*, YLR357W*, YLR372W*, YLR390W-A*, YLR417W*, YLR418C*, YLR442C*, YML001W*, YML036W*, YML062C*, YML097C*, YML121W*, YMR024W*, YMR031C*, YMR031W-A*, YMR070W*, YMR077C*, YMR078C*, YMR080C*, YMR106C*, YMR142C*, YMR179W*, YMR186W*, YMR202W*, YMR223W*, YMR224C*, YMR225C*, YMR263W*, YMR284W*, YNL032W*, YNL072W*, YNL220W*, YNL229C*, YNL243W*, YNL246W*, YNL250W*, YNL281W*, YNR006W*, YOL001W*, YOL004W*, YOL051W*, YOL061W*, YOL068C*, YOL072W*, YOL138C*, YOR003W*, YOR043W*, YOR089C*, YOR123C*, YOR241W*, YOR279C*, YOR321W*, YOR322C*, YOR327C*, YOR368W*, YPL002C*, YPL031C*, YPL041C*, YPL047W*, YPL065W*, YPL084W*, YPL106C*, YPL144W*, YPL161C*, YPL194W*, YPL205C*, YPL240C*, YPL254W*, YPR030W*, YPR049C*, YPR133W-A*, TLC1, YAL043C, YAR007C, YBL041W, YBR002C, YBR136W, YBR167C, YBR192W, YBR256C, YCL004W, YDL029W, YDL060W, YDL097C, YDL120W, YDL147W, YDR021W, YDR037W, YDR041W, YDR054C, YDR060W, YDR118W, YDR246W, YDR394W, YDR396W, YDR397C, YDR404C, YDR412W, YDR413C, YDR499W, YEL034W, YER013W, YER043C, YER157W, YFL037W, YFR052W, YGL044C, YGL099W, YGL120C, YGL150C, YGL169W, YGR002C, YGR065C, YGR074W, YGR075C, YGR091W, YGR099W, YGR158C, YGR198W, YGR280C, YHR058C, YHR070W,

	YHR083W, YHR119W, YHR143W-A, YIL051C, YIL062C, YIL144W, YJL026W, YJL076W, YJL173C, YJR017C, YJR057W, YJR065C, YKL006C-A, YKR022C, YKR038C, YKR083C, YLR071C, YLR103C, YLR106C, YLR355C, YMR061W, YMR076C, YMR277W, YMR301C, YMR314W, YNL132W, YNL137C, YNL261W, YNL306W, YNL312W, YNR035C, YOL133W, YOL146W, YOR074C, YOR117W, YOR160W, YOR335C, YPL083C, YPL128C, YPL153C, YPL204W, YPR103W, YPR108W, YPR144C, YPR175W, YPR178W
**Long** (182)	YAL010C*, YBL006C*, YBL032W*, YBR048W*, YBR111W-A*, YBR134W*, YBR156C*, YBR179C*, YBR233W*, YBR275C*, YBR283C*, YBR284W*, YBR292C*, YBR300C*, YCR020C-A*, YCR020W-B*, YCR026C*, YCR031C*, YCR037C*, YCR047C*, YCR065W*, YCR071C*, YCR073W-A*, YCR081W*, YCR082W*, YCR086W*, YDL083C*, YDL151C*, YDL167C*, YDR017C*, YDR083W*, YDR138W*, YDR156W*, YDR173C*, YDR174W*, YDR184C*, YDR195W*, YDR442W*, YDR443C*, YDR447C*, YDR450W*, YDR470C*, YEL053C*, YER116C*, YER155C*, YGL003C*, YGL039W*, YGL136C*, YGL151W*, YGR118W*, YGR157W*, YGR159C*, YGR160W*, YGR281W*, YHL012W*, YHR013C*, YHR021C*, YHR075C*, YHR203C*, YIL128W*, YIL149C*, YJL138C*, YJL179W*, YJL182C*, YJL190C*, YJR043C*, YJR055W*, YJR079W*, YJR080C*, YJR145C*, YKL113C*, YKL114C*, YKL164C*, YKR057W*, YKR059W*, YLL026W*, YLR032W*, YLR264W*, YLR322W*, YLR337C*, YLR338W*, YLR423C*, YLR453C*, YML024W*, YML034W*, YML060W*, YML061C*, YMR091C*, YMR116C*, YMR125W*, YMR143W*, YMR167W*, YMR269W*, YNL016W*, YNL025C*, YNL064C*, YNL133C*, YNL147W*, YOL020W*, YOL109W*, YOL121C*, YOR008C-A*, YOR040W*, YOR066W*, YOR078W*, YOR144C*, YOR156C*, YOR182C*, YOR183W*, YOR235W*, YOR293W*, YPL017C*, YPL042C*, YPL049C*, YPL068C*, YPL105C*, YPL157W*, YPL178W*, YPL189W*, YPL193W*, YPL213W*, YPL268W*, YPR051W*, YPR070W*, YPR131C*, YPR132W*, YAL038W, YBL035C, YBL074C, YBR049C, YBR088C, YBR109C, YBR154C, YBR254C, YDL102W, YDL163W, YDL164C, YDL165W, YDL208W, YDL220C, YDR082W, YDR331W, YDR498C, YDR510W, YEL019C, YEL032W, YFR005C, YFR037C, YGL201C, YHR042W, YIR008C, YJL005W, YJL031C, YJL032W, YJR022W, YKL022C, YKL165C, YKR008W, YLR010C, YLR167W, YLR175W, YLR197W, YLR198C, YLR229C, YLR230W, YLR316C, YLR317W, YLR339C, YLR340W, YLR424W, YML034C-A, YMR239C, YNL102W, YNL216W, YNL262W, YOR217W, YOR218C, YOR254C, YOR260W, YOR278W, YPL255W, YPR133C
**No Info** (14)	YER177W, YDR099W, YOR033C, YDR110W, YER173W, YDR217C, YPL024W, YDR143C, YGR092W, YHR082C, YNL282W, YLR026C, YKL203C, YDR315C
